# Circulating microRNAs as potential biomarkers for coronary plaque rupture

**DOI:** 10.18632/oncotarget.18308

**Published:** 2017-05-30

**Authors:** Sufang Li, Chongyou Lee, Junxian Song, Changlin Lu, Jun Liu, Yuxia Cui, Huizhu Liang, Chengfu Cao, Feng Zhang, Hong Chen

**Affiliations:** ^1^ Department of Cardiology, Peking University People's Hospital, Beijing, China; ^2^ Beijing Key Laboratory of Early Prediction and Intervention of Acute Myocardial Infarction, Peking University People's Hospital, Beijing, China; ^3^ Center for Cardiovascular Translational Research, Peking University People's Hospital, Beijing, China; ^4^ Department of Cardiology, Beijing Chao-Yang Hospital, Capital Medical University, Beijing, China

**Keywords:** coronary artery disease, plaque rupture, biomarker, diagnosis, microRNA

## Abstract

Coronary plaque rupture is the most common cause of acute coronary syndrome. However, the timely biomarker-based diagnosis of plaque rupture remains a major unmet clinical challenge. Balloon dilatation and stent implantation during percutaneous coronary intervention (PCI) could cause plaque injury and rupture. Here we aimed to assess the possibility of circulating microRNAs (miRNAs) as biomarkers of acute coronary plaque rupture by virtue of the natural model of PCI-induced plaque rupture. Stable coronary artery disease patients underwent PCI with single stent implantation were recruited and a three-phase approach was conducted in the present study: (i) profiling of plasma miRNAs in a group of patients before (0 h) and after balloon dilatation for 1 h (1 h vs. 0 h), (ii) replication of significant miRNAs in the second group of patients (1 h vs. 0 h), (iii) validation of a multi-miRNAs panel in the third group of patients (0.5 h, 1 h vs. 0 h). Out of 24 miRNAs selected for replication, 6 miRNAs remained significantly associated with plaque rupture. In the validation phase, combinations of miR-483-5p and miR-451a showed the highest area under the receiver-operating-characteristic curve (AUC) (0.982; CI: 0.907-0.999) in patients with plaque rupture for 0.5 h; combinations of miR-483-5p and miR-155-5p showed the highest AUC (0.898; CI: 0.790-0.962) after plaque rupture for 1 h. In conclusion, using a profiling-replication-validation model, we identified 3 miRNAs including miR-155-5p, miR-483-5p and miR-451a, which may be biomarkers for the early identification of plaque rupture.

## INTRODUCTION

Coronary plaque rupture is the most common cause of acute coronary syndrome (ACS), including unstable angina (UA), acute myocardial infarction (AMI), and sudden death [1]. The accurate identification of patients with ACS caused by plaque rupture may allow early and preventative treatment to avoid the occurrence of adverse cardiovascular events. However, current diagnostic methods remain unable to determine these high-risk patients who suffered from plaque rupture as soon as possible.

It has been reported that some cytokines, chemokines, growth-factors and enzyme in circulation may be biomarkers of unstable plaque [2], but there are still not definite biomarkers of ruptured plaque, which may be partially due to lacking an appropriate model of plaque rupture. Pathological studies have shown that balloon dilatation could lead to atherosclerotic plaque rupture both in patients underwent percutaneous coronary intervention (PCI) [3–7] and in animal models of atherosclerosis [8, 9]. Optical coherence tomography (OCT) study has also proved that stent implantation could cause plaque injury, and that the frequency of plaque injury did not differ between stable and unstable patients [10]. Therefore, PCI-induced plaque injury could be used to mimic plaque rupture that occurred in patients with coronary artery disease (CAD). Patients underwent PCI may be the natural model to study the biomarkers of plaque rupture.

MicroRNAs (miRNAs) are small evolutionarily conserved, 20 to 22 nt, noncoding RNAs that serve as crucial regulators of a range of molecular signaling pathways involved in atherosclerotic plaque progression and regression [11]. Circulating miRNAs are protected from degradation by binding to transport proteins or by being encapsulated in microvesicles and exosomes. So they can be reliably detected in plasma samples [12, 13]. Accumulating studies have revealed that miRNAs may be useful biomarkers in the diagnosis of atherosclerosis and CAD [14]. Herein, we aimed to determine the early circulating miRNAs biomarkers of plaques rupture in CAD patients by virtue of PCI-induced culprit lesion injury.

## RESULTS

### MiRNAs profiles in plasma from patients with plaque rupture for 1 h

**Figure 1:**
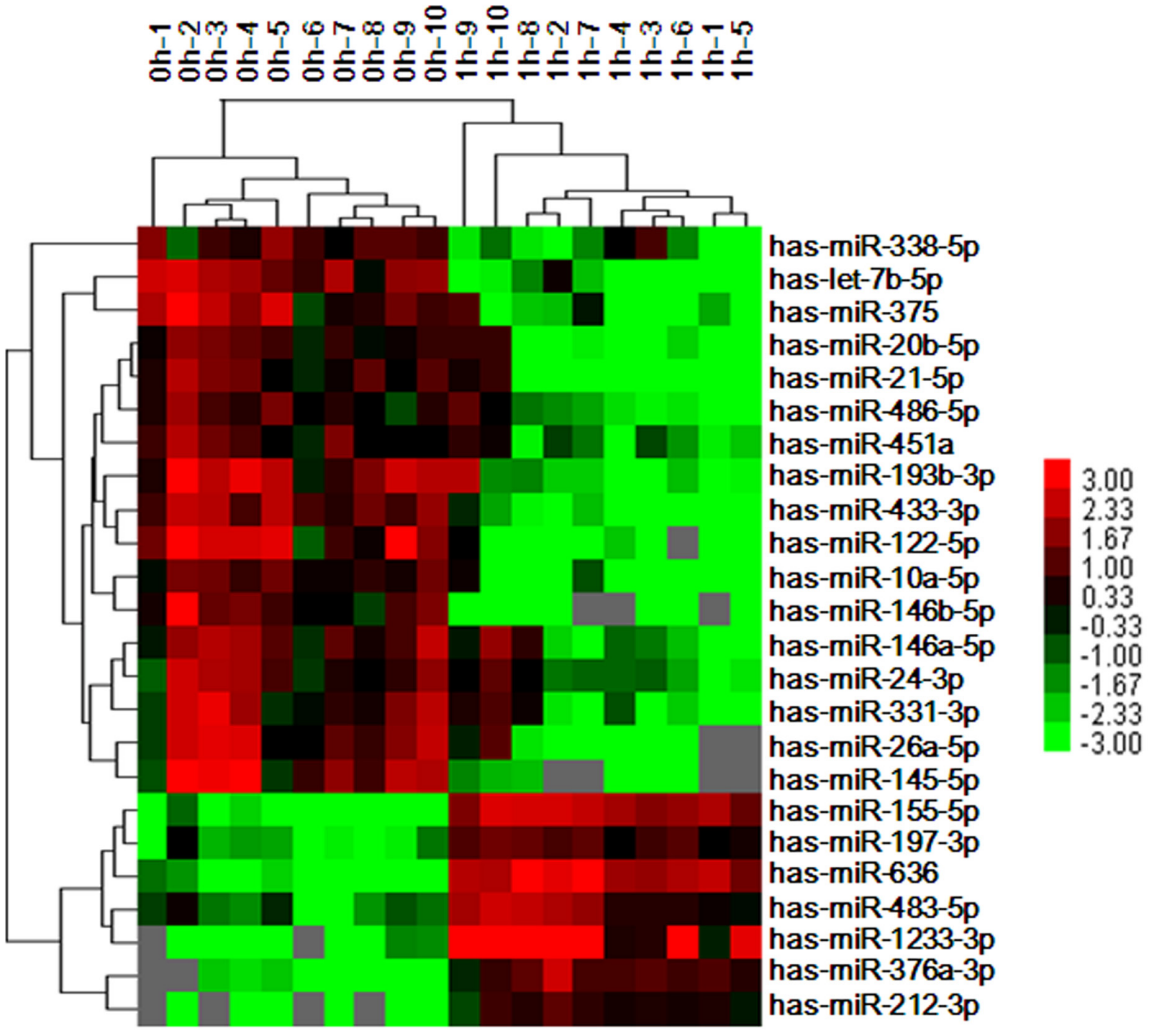
Circulating miRNAs profiles in patients without (0 h) and with plaque rupture for 1 h (1 h) RNA was isolated from EDTA-plasma from patients underwent PCI before (0 h) and after balloon dilatation for 1 h (n=10). The heat map diagram showed the cluster of the differentially expressed circulating miRNAs. MiRNA levels were normalized to the spiked-in miRNA, ath-miR-159a. Red: high expression; green: low expression; gray: undetected.

The clinical characteristics of the study population were presented in Table 1. To determine the effect of plaque rupture on the levels of circulating miRNAs, we compared the levels of 754 miRNAs in plasma samples obtained from 10 patients before (0 h) and after balloon dilatation for 1 h (1 h). The levels of circulating miRNAs after balloon dilatation profoundly differed from the baseline levels (0 h), as illustrated in the heat map diagram (Figure 1). There were 24 differentially expressed miRNAs, including 7 upregulated and 17 downregulated miRNAs, after plaque rupture for 1 h (Table 2).

**Table 1:** Characteristics of the study populations

Variables	Profiling phase	Replication phase		Validation phase
	PCI (n=10)	PCI(n=10)	CAG(n=10)	PCI(n=29)
**Clinical characteristics**				
***Demographics***				
Age (years)	62±10	63±10	62±8	62±12
Male, n (%)	5 (50)	5 (50)	5 (50)	16 (55)
SBP (mm Hg)	132±16	135±18	135±17	131±17
DBP (mm Hg)	81±10	79±5	85±15	77±9
Heart rate (bpm)	67±10	68±8	74±7	71±11
***Coronary artery disease*** *(CAD)* **risk factors**				
Hypertension, n (%)	5 (50)	4 (40)	7 (70)	22 (76)
Diabetes, n (%)	3 (30)	0	2 (20)	9 (31)
Dyslipidemia, n (%)	6 (60)	3 (30)	2 (20)	15 (52)
Smoke, n (%)	3 (30)	4 (40)	4 (30)	14 (48)
Smoke stop>1 year, n (%)	1 (10)	0	1 (10)	3 (10)
Familiar history of CAD, n (%)	1 (10)	3 (30)	2 (20)	8 (28)
**Procedural characteristics (n=10 vessels/group)**				
***Vessel***				
LAD, n (%)	3 (30)	6 (60)	-	16 (55)
LCX, n (%)	2 (20)	2 (20)	-	5 (17)
RCA, n (%)	5 (50)	2 (20)	-	8 (28)
***Other data***				
Maximum stenosis (%)	85.4±6.6	82.0±3.5	66.0±13.5	84.9±6.2
Stent diameter (mm)	3.3±0.6	3.0±0.5	-	3.1±0.5
Stent length (mm)	23.1±6.8	22.8±6.7	-	23.7±6.4
Stent implantation pressure (atm)	11.6±2.3	10.3±2.3	-	9.6±1.3
Predilatation, n (%)	8 (80)	8 (80)	-	24 (83)
Postdilatation, n (%)	10 (100)	9 (90)	-	27 (93)
Largest balloon size for dilatation (mm)	3.5±0.6	3.0±0.6	-	3.1±0.6
Maximum inflation pressure (atm)	14.8±2.1	14.0±2.7	-	14.0±2.8
**Medication**				
Aspirin, n (%)	10 (100)	10 (100)	10 (100)	26 (90)
Clopidogrel, n (%)	8 (80)	8 (80)	10 (100)	21 (72)
Calcium antagonist, n (%)	4 (40)	5 (50)	3 (30)	11 (38)
ACEI, n (%)	1 (10)	2 (20)	1 (10)	7 (24)
ARB, n (%)	2 (20)	2 (20)	4 (40)	8 (28)
β-blocker, n (%)	2 (20)	6 (60)	6 (60)	12 (41)
Statins, n (%)	10 (100)	9 (90)	10 (100)	25 (86)

Data represent mean ± SD. PCI: percutaneous coronary intervention; CAG: coronary artery angiography; SBP: systolic blood pressure; DBP: diastolic blood pressure; LAD: left anterior descending coronary artery; LCX: left circumflex artery; RCA: right coronary artery; ACEI: angiotensin-converting enzyme inhibitor; ARB: angiotensin receptor blocker.

**Table 2:** MiRNAs profiles in the plasma of patients before (0 h) and after balloon dilatation for 1 h (1 h)

No.	Gene ID	Score(d)	Fold change (1 h vs. 0 h)	q-Value (%)
1	hsa-miR-155-5p	9.115	38.403	< 0.0001
2	hsa-miR-212-3p	8.848	25.541	<0.0001
3	hsa-miR-197-3p	7.830	6.465	<0.0001
4	hsa-miR-636	6.724	39.324	<0.0001
5	hsa-miR-483-5p	4.144	6.110	<0.0001
6	hsa-miR-376a-3p	4.141	9.569	<0.0001
7	hsa-miR-1233-3p	3.183	60.650	<0.0001
8	hsa-miR-10a-5p	-6.011	0.126	<0.0001
9	hsa-let-7b-5p	-5.939	0.061	<0.0001
10	hsa-miR-433-3p	-5.678	0.060	<0.0001
11	hsa-miR-20b-5p	-3.945	0.225	<0.0001
12	hsa-miR-21-5p	-3.896	0.166	<0.0001
13	hsa-miR-26a-5p	-3.754	0.107	<0.0001
14	hsa-miR-375	-3.625	0.101	<0.0001
15	hsa-miR-122-5p	-3.294	0.034	<0.0001
16	hsa-miR-451a	-3.254	0.279	<0.0001
17	hsa-miR-486-5p	-3.126	0.298	<0.0001
18	hsa-miR-146a-5p	-3.110	0.280	<0.0001
19	hsa-miR-331-3p	-3.020	0.182	<0.0001
20	hsa-miR-24-3p	-2.944	0.275	<0.0001
21	hsa-miR-193b-3p	-2.918	0.134	<0.0001
22	hsa-miR-146b-5p	-2.840	0.034	<0.0001
23	hsa-miR-145-5p	-2.817	0.055	<0.0001
24	hsa-miR-338-5p	-2.801	0.247	<0.0001

Circulating miRNAs showing significant change in plasma from patients after balloon dilatation for 1 h compared with the baseline levels (0 h) (n = 10). Only miRNAs with more than 2-fold change and false discovery rate <0.0001% are shown here.

### Replication of circulating miRNAs in patients with plaque rupture for 1 h

**Figure 2:**
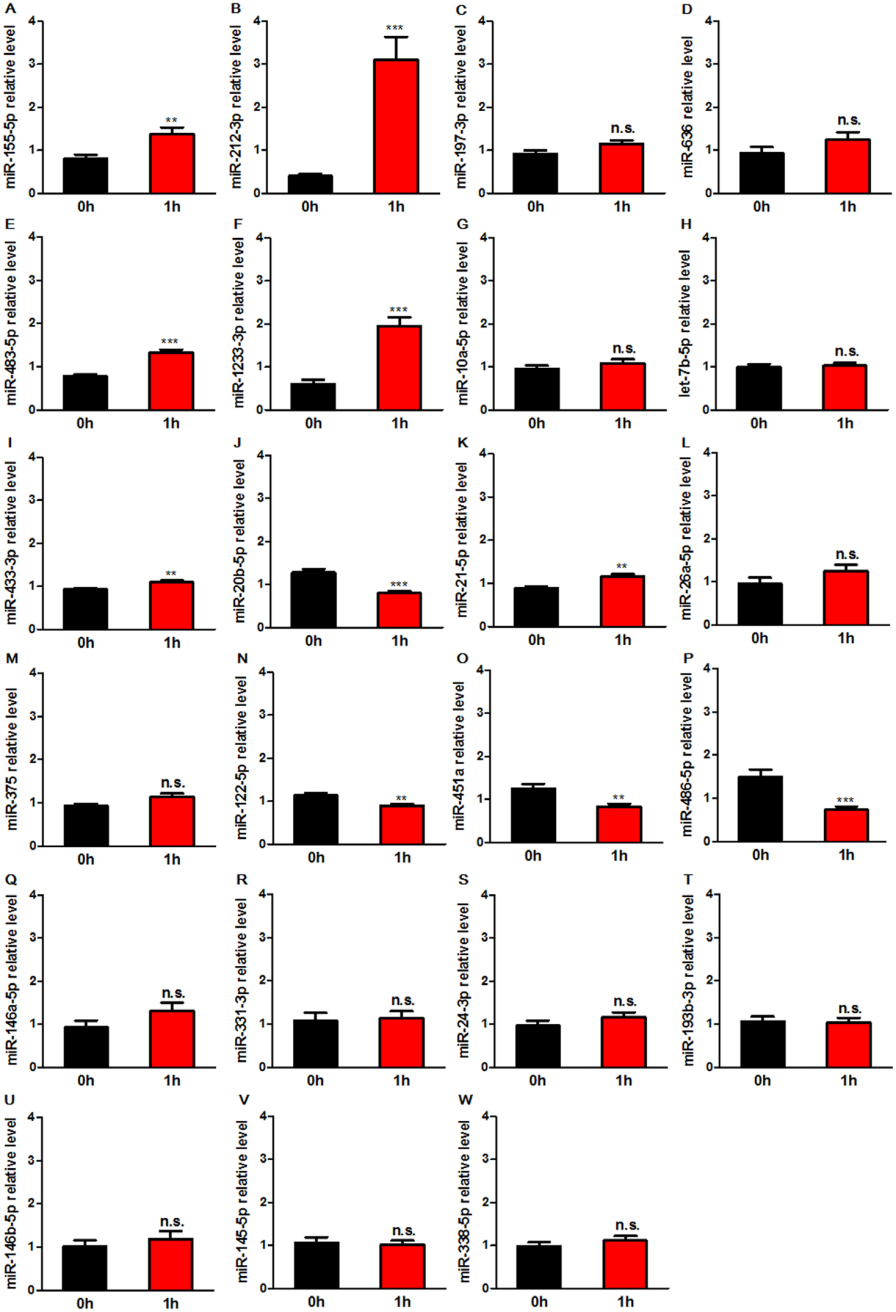
Expression levels of circulating miRNAs in patients before (0 h) and after balloon dilatation for 1 h (1 h) The levels of differentially expressed miRNAs determined by miRNA array were further measured by real-time PCR in separate 10 patients underwent PCI. MiRNA levels were normalized to the spiked-in miRNA, cel-miR-39. Data are expressed as mean ± SEM. n. s.: no significance. **P<0.01, ***P<0.001 vs. 0 h.

To confirm the results obtained by analyzing the miRNAs profiles, the above 24 differentially expressed miRNAs were forwarded into the replication phase. We measured the selected miRNAs levels in separate 10 patients underwent PCI by real-time PCR. The abundance of miR-376a was too low to be detected (data not shown). As shown in Figure 2, eight miRNAs showed significantly differential levels in CAD patients after balloon dilatation for 1 h in both profiling and replication phase. These miRNAs included miR-155-5p, miR-212-3p, miR-483-5p, miR-1233-3p, miR-20b-5p, miR-122-5p, miR-451a, and miR-486-5p.

**Figure 3:**
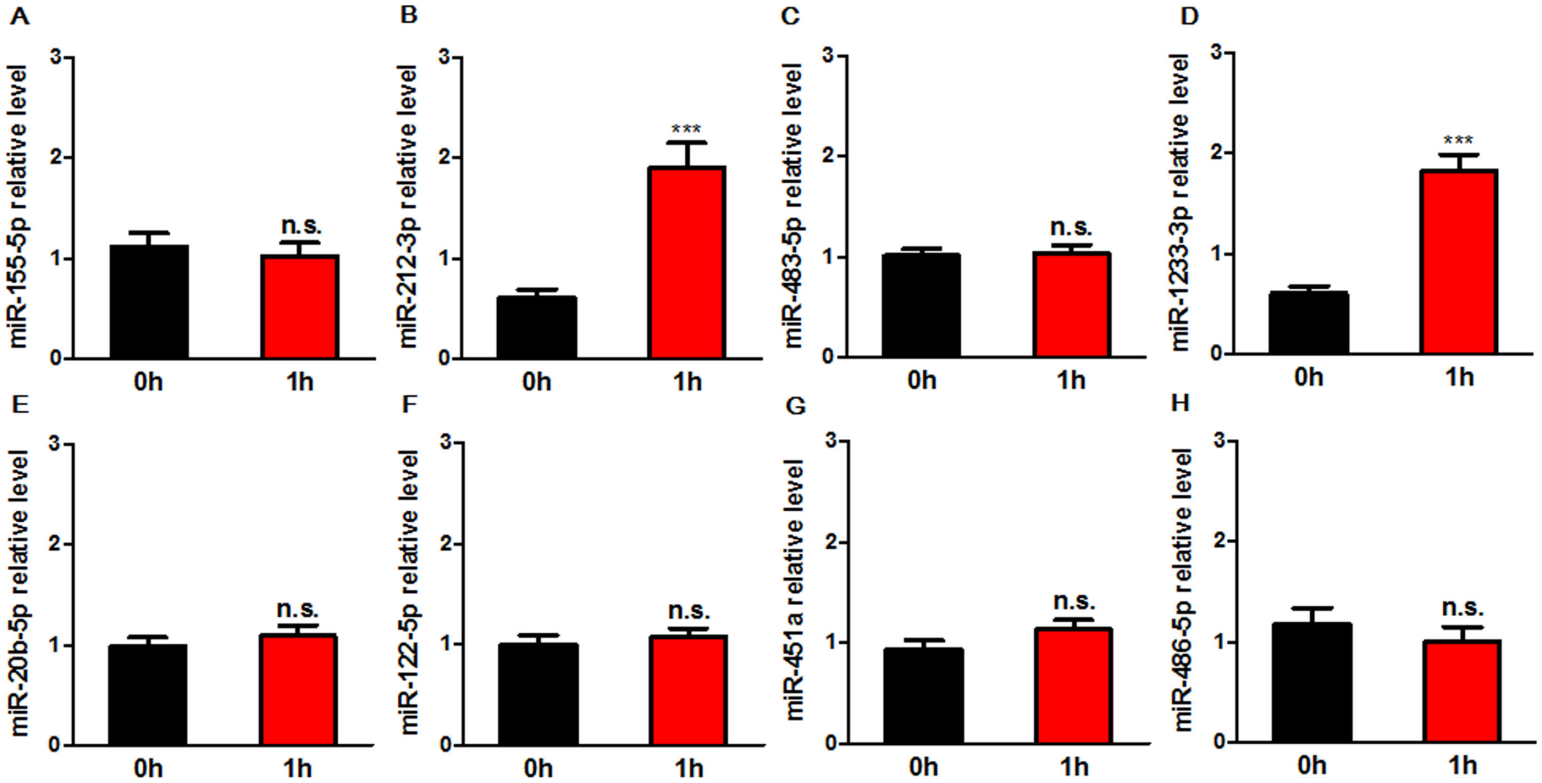
Expression levels of circulating miRNAs in patients before (0 h) and after the end of CAG for 1 h (1 h) The levels of 8 miRNAs selected in replication phase were determined by real-time PCR in 10 patients only underwent CAG. MiRNA levels were normalized to the spiked-in miRNA, cel-miR-39. Data are expressed as mean ± SEM. ***P<0.001 vs. 0 h. n. s.: no significance; CAG: coronary artery angiography.

To exclude the effect of other factors, e.g. contrast agent, on circulating miRNAs, we detected the levels of 8 miRNAs in 10 patients only underwent coronary artery angiography (CAG) at baseline (0 h) and after the end of CAG for 1 h by real-time PCR. Results showed that miR-212-3p and miR-1233-3p were significantly increased after CAG (Figure 3), and the other 6 miRNAs levels were not changed. Therefore, 6 miRNAs including miR-155-5p, miR-483-5p, miR-20b-5p, miR-122-3p, miR-451a, and miR-486-5p were selected for the validation phase.

### Validation of circulating miRNAs in patients with plaque rupture for 0.5 h and 1 h

**Figure 4:**
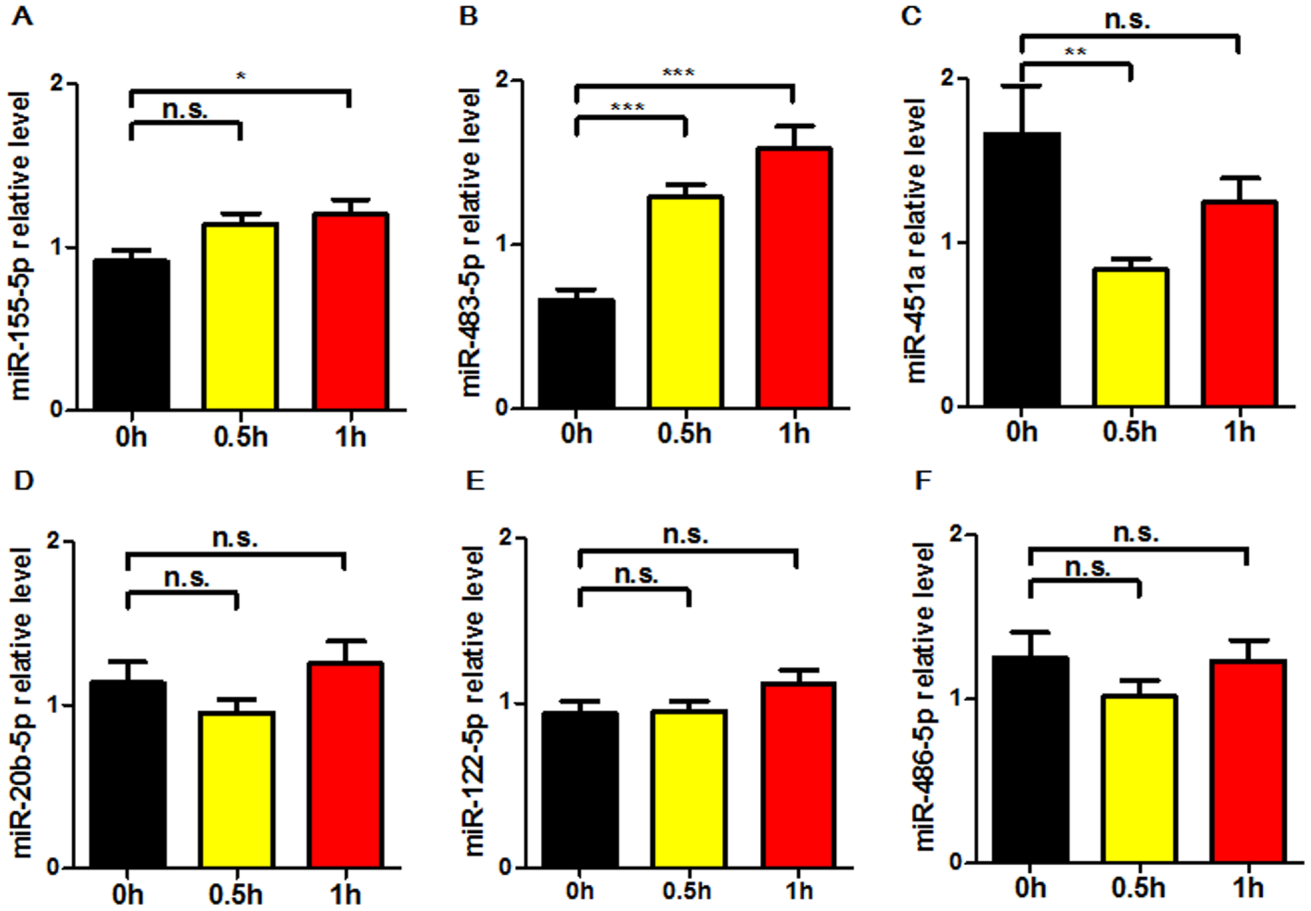
Expression levels of circulating miRNAs in patients before (0 h) and after balloon dilatation for 0.5 h (0.5 h) and 1 h (1 h) The levels of 6 selected miRNAs were further determined by real-time PCR in separate 29 patients underwent PCI. MiRNA levels were normalized to the spiked-in miRNA, cel-miR-39. Data are expressed as mean ± SEM. n. s.: no significance. *P<0.05, **P<0.01, ***P<0.001 vs. 0 h.

To verify the levels of the above 6 miRNAs and to determine the earlier time of differential expression of miRNAs, we further measured these circulating miRNAs in another 29 patients before and after balloon dilatation for 0.5 h and 1 h by real-time PCR. As shown in Figure 4, miR-155-5p and miR-483-5p were upregulated both at 0.5 h and 1 h after balloon dilatation; miR-451a was significantly downregulated only at 0.5 h. By contrast, miR-20b-5p, miR-122-3p and miR-486-5p were not changed at 0.5 h and 1 h compared to 0 h. Therefore, miR-155-5p, miR-483-5p and miR-451a were selected for the analysis of diagnostic accuracy.

### Diagnostic accuracy of selected miRNAs in patients with plaque rupture

**Figure 5:**
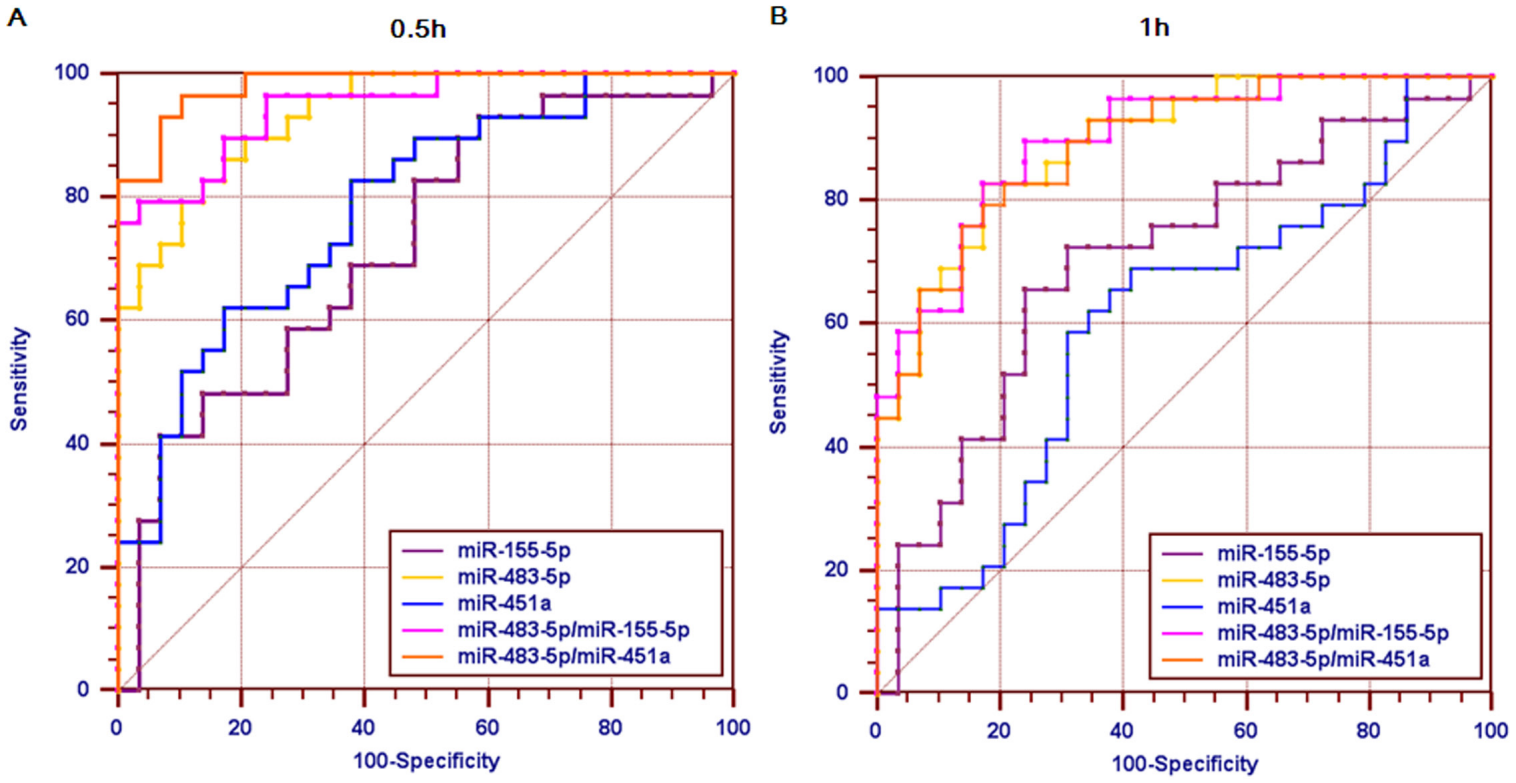
Diagnostic power of circulating miRNAs Receiver operator characteristic (ROC) curves and area under the ROC curve (AUC) are given for single miRNA (miR-155-5p, miR-483-5p, miR-451a) and combinations (miR-483-5p/miR-155-5p, miR-483-5p/miR-451a) to discriminate patients with plaque rupture for 0.5 h **(A)** or 1 h **(B)** from whom with no plaque rupture. ROC curves constructed using 2^-ΔΔCt^ values.

To evaluate the potential diagnostic value of these validated miRNAs, receiver operating characteristic (ROC) curves analyses were constructed and the areas under the curve (AUC) values were determined in 29 patients from the validation phase (Table 3, Figure 5). Among the 3 validated miRNAs, miR-483-5p showed the highest AUC both in patients with PCI-induced plaque rupture for 0.5 h (AUC: 0.937; CI: 0.841-0.984) and 1 h (AUC: 0.894; CI: 0.785-0.960). Combinations of miR-483-5p and miR-451a showed the highest AUC (0.982; CI: 0.907-0.999) in patients with plaque rupture for 0.5 h. Combinations of miR-483-5p and miR-155-5p slightly increased the AUC (0.898; CI: 0.790 - 0.962) at 1 h after plaque rupture compared with that of miR-483-5p alone. Thus, the highest diagnostic accuracy for plaque rupture was achieved by applying the 2-miRNA combination of miR-483-5p and miR-451a at 0.5 h after plaque rupture, and combination of miR-483-5p and miR-155-5p at 1 h.

**Table 3:** Receiver operating characteristic curves

	AUC	95% CI	P-value	Cut-off value	Specificity (%)	Sensitivity (%)
**0.5 h vs. 0 h**						
miR-155-5p	0.723	0.590 - 0.832	0.0009	>0.7935	44.83	89.66
miR-483-5p	0.937	0.841 - 0.984	<0.0001	>0.9342	79.31	89.66
miR-451a	0.785	0.657 - 0.882	<0.0001	≤0.8501	82.76	62.07
miR-483-5p/miR-155-5p	0.948	0.855 - 0.989	<0.0001	>1.4537	**96.55**	79.31
miR-483-5p/miR-451a	0.982	0.907 - 0.999	<0.0001	>0.2387	89.66	**96.55**
**1 h vs. 0 h**						
miR-155-5p	0.705	0.571 - 0.818	0.0027	>0.9644	68.97	72.41
miR-483-5p	0.894	0.785 - 0.960	<0.0001	>0.9813	**82.76**	82.76
miR-451a	0.606	0.469 - 0.732	0.152	≤0.9308	68.97	58.62
miR-483-5p/miR-155-5p	0.898	0.790 - 0.962	<0.0001	>0.6391	75.86	**89.66**
miR-483-5p/miR-451a	0.891	0.781 - 0.957	<0.0001	>1.1161	79.31	82.76

AUC: area under the curve; CI: confidence interval.

## DISCUSSION

MiRNAs were involved in the whole pathological process of atherosclerotic plaque initiation, progression, and rupture [15], and may be useful biomarkers of CAD, including stable CAD [16, 17], UA [18–20], AMI [21–23], and so on. To date, no specific biomarker diagnosis is available for identifying patients with plaque rupture. In the present study, we provide evidence for the first time that circulating miRNAs may serve as novel diagnostic biomarkers in CAD patients with plaque rupture. Because balloon dilatation and stent implantation could cause plaque injury, patients underwent PCI were selected as the natural model of plaque rupture in the study.

We performed profiling, replication and validation of circulating miRNAs that are possible biomarkers for the early diagnosis of coronary plaque rupture. Three major findings were observed: firstly, three miRNAs were differently expressed after plaque rupture: miR-155-5p and miR-483-5p were upregulated after plaque rupture for 0.5 h and 1 h, miR-451a was downregulated only after plaque rupture for 0.5 h. Secondly, of the three miRNAs, miR-483-5p showed the highest discriminatory power in the early diagnosis of plaque rupture for 0.5 h (AUC: 0.937; CI: 0.841-0.984) and 1 h (AUC: 0.894; CI: 0.785-0.960). Thirdly, combinations of miR-483-5p and miR-451a showed the highest discriminatory power in patients with plaque rupture for 0.5 h (AUC: 0.982; CI: 0.907-0.999). The discriminatory power was not obviously improved by combining miR-483-5p and miR-155-5p in patients with plaque rupture for 1 h (AUC: 0.898; CI: 0.790-0.962).

MiR-155 was reported to be closely related with inflammation and was a pleiotropic molecule in regulating atherosclerosis [24]. Jahantigh M et al. found that miR-155 was upregulated in human carotid plaque samples, which could prompt the inflammatory response during atherosclerosis by repressing B-cell leukemia/lymphoma 6 (Bcl6) in macrophages [25]. The levels of miR-155 were increased in CD14^+^ monocytes from patients with CAD, which was correlated with the elevation of pro-atherosclerotic cytokine tumor necrosis factors (TNF-α) and interleukin (IL-6) [26]. The serum levels of miR-155 were also higher in post-AMI patients who experienced cardiac death within 1 year after discharge than those who did not experience any cardiovascular events [27]. These findings suggested that the upregulation of circulating miR-155 induced by plaque rupture in our study may play an adverse role in CAD patients by promoting inflammatory response. Nevertheless, there were opposite reports referring to the effects of miR-155 in CAD. Li et al. thought that increased miR-155 in patients with atherosclerosis may play a protective role during foam cell formation by targeting calcium-regulated heat stable protein 1 (CARHSP1) [28]. Another study also showed that upregulated miR-155 in patients with CAD may inhibit atherosclerosis progression via targeting mitogen-activated protein kinase kinase kinase 10 (MAP3K10) [29]. Therefore, the definite effects of miR-155 in CAD patients with plaque rupture remain to be further confirmed by clinical and basic research.

MiR-451a is highly conserved in evolution and is expressed mostly in blood and heart [30]. MiR-451a was found to be upregulated in human infarcted hearts with less than 7 days after infarction compared to healthy adult hearts [31]. Another research group showed that miR-451a protected against ischemia/reperfusion-induced cardiomyocyte death by targeting CUG triplet repeat-binding protein2 (CUGBP2)-cyclooxygenase-2 (COX-2) pathway [32]. They also found that miR-451a was responsible for ischemic preconditioning-induced cardioprotective effects via targeting Rac-1-mediated oxidative stress signaling pathway [33]. Furthermore, overexpression of miR-451a could suppress cardiac hypertrophy and autophagy by targeting tuberous sclerosis complex 1 (TSC1) [34]. Collectively, these data suggested that the transient reduction of miR-451a observed in our study may represent an adverse effect in CAD patients with plaque rupture, which may be harmful to myocardial survival.

MiR-483-5p is a conserved sequence transcribed with its host gene, insulin-like growth factor 2 (IGF2) [35]. However, unlike IGF2, miR-483-5p was found to inhibit angiogenesis *in vitro* by targeting serum response factor (SRF), which provided a clue for combating angiogenesis in CAD patients [36]. Meanwhile, miR-483-5p was observed to suppress the proliferation of glioma cells via directly targeting extracellular signal-regulated kinase 1 (ERK1) [37]. Furthermore, miR-483-5p and miR-483-3p could cooperatively target two pro-fibrosis factors, platelet-derived growth factor-β (PDGF-β) and tissue inhibitor of metalloproteinase 2 (TIMP2) in liver [38]. Taken together, these findings implied that the upregulation of plasma miR-483-5p may be beneficial for CAD patients to resist a series of adverse effects caused by plaque rupture.

Heparin could inhibit the activity of reverse transcriptase and DNA polymerase in PCR. As our samples were from patients injected with heparin during PCI, we added heparinase into the RNA samples before PCR to eliminate the effect of heparin on PCR [39]. By determining the levels of differentially expressed miRNAs in CAD patients only underwent CAG, we excluded miR-212-3p and miR-1233-3p. The upregulation of these two miRNAs may be induced by contrast agent. This study is limited by the relatively small sample size and by lack of imaging data to show plaque rupture. Larger studies and imaging evidences are needed to confirm the diagnostic capacity of identified miRNAs on plaque rupture. In addition, whether differentially expressed circulating miRNAs is a result of different release mechanisms or is a source of regulated intracellular production, which remains to be determined in future studies.

In conclusion, using PCI-induced plaque injury that mimics the plaque rupture, we identified 3 miRNAs, miR-155-5p, miR-483-5p and miR-451a, which may provide clinically useful information for the early diagnosis of CAD patients with plaque rupture.

## MATERIALS AND METHODS

### Study population

This study was performed in accordance with the Helsinki declaration and was approved by the ethics review board of Peking University People's Hospital. All the enrolled patients completed their written informed consent. Stable CAD patients underwent PCI with single stent implantation or only underwent CAG were included in the study. Criteria for the diagnosis of CAD were according to the European Society of Cardiology guideline [40]. Clinical exclusion criteria were (1) AMI or coronary artery bypass grafting (CABG) within 4 weeks, (2) cardiac troponin I (TNI) ≧0.04 ng/ml and/or creatine kinase (CK-MB) ≧5 ng/ml before PCI, (3) severe hepatic or renal dysfunction, (4) chronic or ongoing inflammatory diseases.

### Study design

The overall study concept consisted of 3 phases including 59 patients with stable CAD: profiling phase, replication phase and validation phase. (1) The profiling phase consisted of 10 patients underwent PCI (1 h vs. 0 h): the RNA samples from plasma collected before (0 h) and after balloon dilatation for 1 h (1 h) were considered as control (0 h) and plaque rupture group (1 h), respectively. In the profiling phase, 754 human miRNAs were assayed, and out of those, 24 miRNAs were selected to enter the replication phase for real-time PCR detection. (2) The replication phase consisted of separate 20 patients, of whom 10 underwent PCI and 10 only underwent CAG (negative control) (1 h vs. 0 h): out of the 24 miRNAs replicated in this phase, 6 entered the validation phase. (3) The validation phase consisted of another 29 patients underwent PCI (0.5 h, 1 h vs. 0 h). The selected 6 miRNAs were further tested by real-time PCR. The significantly differential expressed miRNAs at 0.5 h and 1 h after balloon dilatation versus 0 h (before balloon dilatation) were used to determine the diagnostic potential for plaque rupture.

### Blood collection

Venous blood samples were collected into EDTA-containing tubes and were processed for isolation of plasma within 4 h after collection. The plasma was centrifuged at 3000 rpm for 10 min at 4 °C and frozen at -80 °C until assayed. Blood from patients underwent PCI was collected before (0 h) and after balloon dilatation for 0.5 h and 1 h. Blood from patients only underwent CAG was collected before (0 h) and after the end of CAG for 1 h. If there were no predilatation during PCI, the time of stent expansion was considered as the onset time of plaque rupture.

### Isolation of circulating RNA from plasma

Total RNA was isolated from human plasma with miRNeasy Serum/Plasma Kit (Qiagen, Hilden, Germany) according to the manufactures’ recommendations. 200 μl plasma were lysed with 1000 μl Qiazol and incubated for 5 min at room temperature to ensure complete dissociation of nucleoprotein complexes. Subsequently, 200 μl chloroform were added and the mixture was shaken vigorously for 15 s. After 5 min at room temperature, the mixture was centrifuged for 15 min at 12000 g and 4 °C. The upperaqueous phase was transferred to a fresh reagent tube and mixed with 1.5 volumes of ethanol. Purification of RNA was performed using the miRNeasy Serum/Plasma Kit. RNA was eluted in 14 μl RNAse-free water. Each plasma sample was supplemented with 10 fmol ath-miR-159a (for array) or *Caenorhabditis elegans* miR-39 (cel-miR-39) (for real-time PCR) after addition of Qiazol as a spiked-in control to normalize for individual RNA-isolation-related variations.

### miRNAs array

For miRNAs profiles, total RNA from plasma was analysed using the low density TaqMan Human MicroRNA Card A (version 2.0) and B (version 3.0) Array (Applied Biosystems, Foster City, CA) according to the manufacturer's protocol. Each array card set contains a total of 384 TaqMan miRNA assays and the two array cards enable assaying of 754 human miRNAs. Briefly, approximately 30 ng of total RNA were reverse-transcribed to cDNA with Taqman miRNA reverse transcription kit and Megaplex™ RT Primers (Human Pool A and Pool B, Applied Biosystems) followed by a pre-amplification step using TaqMan^®^PreAmp Master Mix and Megaplex^™^ PreAmp Primers (Human Pool A and Pool B). Subsequently, real-time PCR amplification of miRNAs using TaqMan MicroRNA Array (card A and B) was performed on an Applied Biosystem ViiA™ 7 Real-Time PCR System. The RNA samples were treated with heparinase (0.3 U/sample) during the process of reverse transcription to minimize the effect of heparin on the miRNAs measurements [39].

The miRNAs with cycle threshold (Ct) <40 in at least 16 out of 20 samples (0 h n=10, 1 h n=10) for card A and B were considered as expressed. Raw data were analyzed using Data Assist software for TaqMan gene expression assays version 3.0 (Applied Biosystems). The miRNA expression was normalized to the ath-miR-159a, a spiked-in control on the array card. To identify differentially expressed miRNAs, the data were subjected to significance analysis of microarrays (SAM). The miRNAs that showed at least 2-fold change and a q-value <0.0001% were considered to be differentially expressed.

### Real-time PCR

For quantification of differentially expressed miRNAs, a fixed volume of diluted RNA (5 μl, 10 ng) treated with 0.3U heparinase [39] was subjected to reverse transcription using the TaqMan^®^ MicroRNA Reverse Transcription Kit (Applied Biosystems, Foster City, CA). Subsequently, 1.33 μl of the product was amplified using the TaqMan Universal PCR Master Mix, with AmpErase UNG and miRNA-specific stem-loop primers (Applied Biosystems, Foster City, CA) for the corresponding miRNA. Real-time PCR reactions were performed on an Applied Biosystem ViiA™ 7 Real-Time PCR System. Ct ≥40 was considered as undetermined. Ct values were normalized to cel-miR-39 and the miRNA level was expressed as 2^-ΔΔCt^.

### Statistical analysis

The quantitative data were represented as the mean ± standard deviation (SD) or standard error of the mean (SEM). Quantitative data were analyzed using one-way ANOVA followed by the Tukey multiple-comparisons test or Student's t-test for the comparison of two groups. For categorical variables, the chi-square test was used. ROC curves and AUC were computed to assess specificity and sensitivity of single-plasma miRNAs and their combination via binary logistic regression analysis. All tests were 2-sided and P values < 0.05 were considered to be statistically significant. SPSS 17.0, Medcalc12.5.0.0 and Graphpad Prism 5 were used for the statistical analysis.

## Abbreviations

ACS: acute coronary syndrome
PCI: percutaneous coronary intervention
CAD: coronary artery disease
miRNA: microRNA
PCR: polymerase chain reaction
ROC: receiver operating characteristic
AUC: area under the ROC curve
CI: confidence interval
UA: unstable angina
AMI: acute myocardial infarction
CAG: coronary artery angiography.

## References

[1] Falk E, Nakano M, Bentzon JF, Finn AV, Virmani R (2013). Update on acute coronary syndromes: the pathologists' view. Eur Heart J.

[2] Mueller C (2014). Biomarkers and acute coronary syndromes: an update. Eur Heart J.

[3] Mizuno K, Kurita A, Imazeki N (1984). Pathological findings after percutaneous transluminal coronary angioplasty. Br Heart J.

[4] Soward AL, Essed CE, Serruys PW (1985). Coronary arterial findings after accidental death immediately after successful percutaneous transluminal coronary angioplasty. Am J Cardiol.

[5] Block PC, Myler RK, Stertzer S, Fallon JT (1981). Morphology after transluminal angioplasty in human beings. N Engl J Med.

[6] Hoshino T, Yoshida H, Takayama S, Iwase T, Sakata K, Shingu T, Yokoyama S, Mori N, Kaburagi T (1987). Significance of intimal tears in the mechanism of luminal enlargement in percutaneous transluminal coronary angioplasty: correlation of histologic and angiographic findings in postmortem human hearts. Am Heart J.

[7] Angelini A, Rubartelli P, Mistrorigo F, Della Barbera M, Abbadessa F, Vischi M, Thiene G, Chierchia S (2004). Distal protection with a filter device during coronary stenting in patients with stable and unstable angina. Circulation.

[8] Block PC, Baughman KL, Pasternak RC, Fallon JT (1980). Transluminal angioplasty: correlation of morphologic and angiographic findings in an experimental model. Circulation.

[9] Sanborn TA, Faxon DP, Waugh D, Small DM, Haudenschild C, Gottsman SB, Ryan TJ (1982). Transluminal angioplasty in experimental atherosclerosis. Analysis for embolization using an in vivo perfusion system. Circulation.

[10] Gonzalo N, Serruys PW, Okamura T, Shen ZJ, Onuma Y, Garcia-Garcia HM, Sarno G, Schultz C, van Geuns RJ, Ligthart J, Regar E (2009). Optical coherence tomography assessment of the acute effects of stent implantation on the vessel wall: a systematic quantitative approach. Heart.

[11] Feinberg MW, Moore KJ (2016). MicroRNA regulation of atherosclerosis. Circ Res.

[12] Mitchell PS, Parkin RK, Kroh EM, Fritz BR, Wyman SK, Pogosova-Agadjanyan EL, Peterson A, Noteboom J, O'Briant KC, Allen A, Lin DW, Urban N, Drescher CW (2008). Circulating microRNAs as stable blood-based markers for cancer detection. Proc Natl Acad Sci U S A;.

[13] Creemers EE, Tijsen AJ, Pinto YM (2012). Circulating microRNAs: novel biomarkers and extracellular communicators in cardiovascular disease?. Circ Res.

[14] Economou EK, Oikonomou E, Siasos G, Papageorgiou N, Tsalamandris S, Mourouzis K, Papaioanou S, Tousoulis D (2015). The role of microRNAs in coronary artery disease: from pathophysiology to diagnosis and treatment. Atherosclerosis.

[15] Andreou I, Sun X, Stone PH, Edelman ER, Feinberg MW (2015). miRNAs in atherosclerotic plaque initiation, progression, and rupture. Trends Mol Med.

[16] Fichtlscherer S, De Rosa S, Fox H, Schwietz T, Fischer A, Liebetrau C, Weber M, Hamm CW, Röxe T, Müller-Ardogan M, Bonauer A, Zeiher AM, Dimmeler S (2010). Circulating microRNAs in patients with coronary artery disease. Circ Res.

[17] D'Alessandra Y, Carena MC, Spazzafumo L, Martinelli F, Bassetti B, Devanna P, Rubino M, Marenzi G, Colombo GI, Achilli F, Maggiolini S, Capogrossi MC, Pompilio G (2013). Diagnostic potential of plasmatic MicroRNA signatures in stable and unstable angina. PLoS One.

[18] Ren J, Zhang J, Xu N, Han G, Geng Q, Song J, Li S, Zhao J, Chen H (2013). Signature of circulating microRNAs as potential biomarkers in vulnerable coronary artery disease. PLoS One.

[19] Zeller T, Keller T, Ojeda F, Reichlin T, Twerenbold R, Tzikas S, Wild PS, Reiter M, Czyz E, Lackner KJ, Munzel T, Mueller C, Blankenberg S (2014). Assessment of microRNAs in patients with unstable angina pectoris. Eur Heart J.

[20] Li S, Sun YN, Zhou YT, Zhang CL, Lu F, Liu J, Shang XM (2016). Screening and identification of microRNA involved in unstable angina using gene-chip analysis. Exp Ther Med.

[21] Liebetrau C, Möllmann H, Dörr O, Szardien S, Troidl C, Willmer M, Voss S, Gaede L, Rixe J, Rolf A, Hamm C, Nef H (2013). Release kinetics of circulating muscle-enriched microRNAs in patients undergoing transcoronary ablation of septal hypertrophy. J Am Coll Cardiol.

[22] Cheng C, Wang Q, You W, Chen M, Xia J (2014). MiRNAs as biomarkers of myocardial infarction: a meta-analysis. PLoS One.

[23] Devaux Y, Mueller M, Haaf P, Goretti E, Twerenbold R, Zangrando J, Vausort M, Reichlin T, Wildi K, Moehring B, Wagner DR, Mueller C (2015). Diagnostic and prognostic value of circulating microRNAs in patients with acute chest pain. J Intern Med.

[24] Xiaoyan W, Pais EM, Lan L, Jingrui C, Lin M, Fordjour PA, Guanwei F (2017). MicroRNA-155: a novel armamentarium against inflammatory diseases. Inflammation.

[25] Nazari-Jahantigh M, Wei Y, Noels H, Akhtar S, Zhou Z, Koenen RR, Heyll K, Gremse F, Kiessling F, Grommes J, Weber C, Schober A (2012). MicroRNA-155 promotes atherosclerosis by repressing Bcl6 in macrophages. J Clin Invest.

[26] Tian FJ, An LN, Wang GK, Zhu JQ, Li Q, Zhang YY, Zeng A, Zou J, Zhu RF, Han XS, Shen N, Yang HT, Zhao XX (2014). Elevated microRNA-155 promotes foam cell formation by targeting HBP1 in atherogenesis. Cardiovasc Res.

[27] Matsumoto S, Sakata Y, Nakatani D, Suna S, Mizuno H, Shimizu M, Usami M, Sasaki T, Sato H, Kawahara Y, Hamasaki T, Nanto S, Hori M, Komuro I (2012). A subset of circulating microRNAs are predictive for cardiac death after discharge for acute myocardial infarction. Biochem Biophys Res Commun.

[28] Li X, Kong D, Chen H, Liu S, Hu H, Wu T, Wang J, Chen W, Ning Y, Li Y, Lu Z (2016). miR-155 acts as an anti-inflammatory factor in atherosclerosis-associated foam cell formation by repressing calcium-regulated heat stable protein 1. Sci Rep.

[29] Zhu J, Chen T, Yang L, Li Z, Wong MM, Zheng X, Pan X, Zhang L, Yan H (2012). Regulation of microRNA-155 in atherosclerotic inflammatory responses by targeting MAP3K10. PLoS One.

[30] Dore LC, Amigo JD, Dos Santos CO, Zhang Z, Gai X, Tobias JW, Yu D, Klein AM, Dorman C, Wu W, Hardison RC, Paw BH, Weiss MJ (2008). A GATA-1-regulated microRNA locus essential for erythropoiesis. Proc Natl Acad Sci U S A.

[31] Bostjancic E, Zidar N, Glavac D (2009). MicroRNA microarray expression profiling in human myocardial infarction. Dis Markers.

[32] Zhang X, Wang X, Zhu H, Zhu C, Wang Y, Pu WT, Jegga AG, Fan GC (2010). Synergistic effects of the GATA-4-mediated miR-144/451 cluster in protection against simulated ischemia/reperfusion-induced cardiomyocyte death. J Mol Cell Cardiol.

[33] Wang X, Zhu H, Zhang X, Liu Y, Chen J, Medvedovic M, Li H, Weiss MJ, Ren X, Fan GC (2012). Loss of the miR-144/451 cluster impairs ischaemic preconditioning-mediated cardioprotection by targeting Rac-1. Cardiovasc Res.

[34] Song L, Su M, Wang S, Zou Y, Wang X, Wang Y, Cui H, Zhao P, Hui R, Wang J (2014). MiR-451 is decreased in hypertrophic cardiomyopathy and regulates autophagy by targeting TSC1. J Cell Mol Med.

[35] Lutter D, Marr C, Krumsiek J, Lang EW, Theis FJ (2010). Intronic microRNAs support their host genes by mediating synergistic and antagonistic regulatory effects. BMC Genomics.

[36] Qiao Y, Ma N, Wang X, Hui Y, Li F, Xiang Y, Zhou J, Zou C, Jin J, Lv G, Jin H, Gao X (2011). MiR-483-5p controls angiogenesis in vitro and targets serum response factor. FEBS Lett.

[37] Wang L, Shi M, Hou S, Ding B, Liu L, Ji X, Zhang J, Deng Y (2012). MiR-483-5p suppresses the proliferation of glioma cells via directly targeting ERK1. FEBS Lett.

[38] Li F, Ma N, Zhao R, Wu G, Zhang Y, Qiao Y, Han D, Xu Y, Xiang Y, Yan B, Jin J, Lv G, Wang L (2014). Overexpression of miR-483-5p/3p cooperate to inhibit mouse liver fibrosis by suppressing the TGF-β stimulated HSCs in transgenic mice. J Cell Mol Med.

[39] Li S, Chen H, Song J, Lee C, Geng Q (2016). Avoiding heparin inhibition in circulating MicroRNAs amplification. Int J Cardiol.

[40] Montalescot G, Sechtem U, Achenbach S, Andreotti F, Arden C, Budaj A, Bugiardini R, Crea F, Cuisset T, Di Mario C, Ferreira JR, Gersh BJ, Task Force Members (2013). 2013 ESC guidelines on the management of stable coronary artery disease: the Task Force on the management of stable coronary artery disease of the European Society of Cardiology. Eur Heart J.

